# Eight-year follow-up of phenotypic progression in a Chinese XLRP pedigree with a novel *RP2* gene mutation

**DOI:** 10.3389/fgene.2026.1783270

**Published:** 2026-05-25

**Authors:** Huihui Sun, Jindou Shi, Suling Yang, Haixia Tian, Xiaoge Yang, Gaifen Xie, Yunshuo Wei, Jiancang Wang

**Affiliations:** 1 Department of Ophthalmology, The Children’s Hospital of Hebei Province, Hebei Provincial Clinical Research Center for Child Health and Disease, Shijiazhuang, China; 2 Department of Imaging, Hebei General Hospital, Hebei Medical University, Shijiazhuang, China

**Keywords:** retinitis pigmentosa, *RP2*, female carrier, phentypic spectra, longitudinal progression

## Abstract

**Background:**

This study characterizes a Chinese X-linked retinitis pigmentosa (XLRP) pedigree harboring a pathogenic variant of the *RP2* gene and presents the findings from 8-years follow-up with the aim of exploring the genotypic and phenotypic spectra.

**Methods:**

We collected data from a Chinese pedigree comprising 23 individuals, including six affected males and five female carriers; all individuals underwent molecular analyses and comprehensive ophthalmic evaluations.

**Results:**

We identified a novel c.392G>A (p.C131Y) mutation of the *RP2* gene*.* The baseline clinical evaluations of four affected males in the average age range of 1–8 years showed visual impairment; the mean visual acuity had a logMAR value of 0.5235 (range: 0.301–0.698) with an average spherical equivalent of −4.15D (range: −3.125D to −6.25D). The funduscopic observations were consistent with typical X-linked retinoschisis presentations, including pigmentary abnormalities, tessellated changes, and yellowish-white punctate exudations. We also detected disorganization of the outer retinal layer and decline of the electroretinography (ERG) amplitudes. Meanwhile, the five heterozygous carriers showed a wide range of phenotypic variability accompanied by mild or moderate visual changes. Intriguingly, asymmetric changes of the fundus were also detected in the retina of one of the carriers. During the 8-year follow-up period, the visual acuity remained unchanged or even improved slightly. Although early-onset myopia was more common in children, there was a slightly increasing trend in annual progression; moreover, while the retinal structure did not differ statistically significantly, the ERG changes indicated a steadily decreasing trend.

**Conclusion:**

The findings of this study provide insights into the pathogenicity of *RP2-*related XLRP, expand the spectrum of disease mutations, and enrich knowledge regarding the clinical phenotypes while highlighting the progressive nature and phenotypic variability.

## Introduction

Inherited retinal degenerations represent a genetically heterogeneous group of disorders characterized by progressive photoreceptor dysfunction and vision loss (Scholl et al., 2016). Among the different types of retinitis pigmentosa (RP), nyctalopia typically presents within the first decade of life, followed by progressive loss of peripheral vision and eventual loss of central vision, often culminating in complete blindness by the age of 40–50 years (Duncan et al., 2007). The main features of RP include progressive loss of visual field (VF), attenuation of the retinal vessels, presence of bone spicule deposits in the fundus, and reduced or non-detectable a-waves and b-waves on the electroretinogram examination.

More than 60 genes have been linked with non-syndromic RP, and these can be inherited in autosomal dominant, recessive, or X-linked recessive pathways. X-linked retinitis pigmentosa (XLRP) is a severe form of RP that accounts for almost 10%–20% of all cases of RP and typically manifests with severe phenotypes in males and variable expressivity in female carriers (Wensel et al., 2021). The retinitis pigmentosa 2 (*RP2*) gene ranks as the second most-frequent cause of XLRP after *RPGR*, and its variants are responsible for approximately 5%–20% of all cases of XLRP (Thompson et al., 2000). Briefly, *RP2* encodes a specific microtubule protein linked to photoreceptors that plays a crucial role in maintaining structure and function (Vingolo et al., 2024). At present, *RP2* mutations have been found to result in more severe forms of retinal degeneration, but there is a lack of clear genotype–phenotype correlations and poor understanding of the mechanisms underlying retinal degeneration in humans (Testa et al., 2025; Veleri et al., 2015). On the one hand, the overlap of several symptoms complicates the ability to differentiate between *RP2-*based and other XLRP-related mutations. On the other hand, it is difficult to predict disease severity on the sole basis of genetic mutations.

Although existing animal models (such as the *RP2*-knockout mice) offer valuable insights into the progression of this disease, these models do not fully replicate the severe phenotypes observed in patients (Veleri et al., 2015). Moreover, earlier studies that established the genetic basis of XLRP have typically focused on *RPGR*, while longitudinal phenotypic characterizations remain limited, particularly for specific *RP2* variants (Testa et al., 2025). It is notable that no targeted or efficient treatments have been developed for *RP2*-related XLRP to date. Therefore, our findings characterizing a novel *RP2* variant in a Chinese XLRP pedigree quantify the baseline phenotypic features using multimodal assessments and establish some longitudinal progression metrics over the follow-up period of 8 years. Our study contributes to the growing body of knowledge on genotype–phenotype correlations and provides some essential benchmarks for clinical trial endpoints and therapeutic windows, particularly for emerging gene therapies targeting *RP2* (Silva et al., 2021). Moreover, the observed phenotypic variability in female carriers challenges conventional XLRP diagnostic paradigms to introduce novel insights regarding carrier phenotypes and disease progression dynamics (Gocuk et al., 2023).

## Methods

### Study design and participant enrollment

The present study employed a longitudinal observational design to characterize the phenotypic progression of XLRP in a Chinese pedigree harboring a pathogenic *RP2* variant. A total of 23 individuals were enrolled in the study, comprising six affected males, five female carriers, and 12 unaffected family members serving as controls. The studies involving human participants were reviewed and approved by the Ethics Committee of Children’s Hospital of Hebei Province. Written informed consent for participation in this study was provided by the patients or their parents/legal guardians/next of kin.

### Genetic analysis

Genomic DNA from the peripheral blood leukocytes of the individuals were subjected to whole-exome sequencing (WES) on an Illumina NovaSeq 6000 platform, and the average depth of coverage was about 100×. Briefly, the data were aligned to hg38/GRCh38, and any single-nucleotide polymorphisms (SNPs), copy number variations (CNVs), and insertions and deletions (indels) were identified. The variants were screened and evaluated using the dbSNP, 1000 Genomes, ClinVar, and Exome Aggregation Consortium databases. The pathogenicity of the mutations was assessed according to the guidelines of the American College of Medical Genetics and Genomics (ACMG). First, the benign or likely-benign mutations were filtered out, and the primers needed to amplify the fragments harboring the candidate variants were designed using Primer3. Then, SIFT, PolyPhen-2, MutationTaster, PROVEAN, and REVEL were used to evaluate the potential pathogenicities of the variants with the default parameters. Next, the sequence alignments of different species were analyzed using ClustalW. Lastly, the three-dimensional models of the proteins were generated using SWISS-MODEL.

### Clinical assessments

Comprehensive ophthalmic evaluations were conducted, including best-corrected visual acuity (BCVA) measurements using the ETDRS chart, cycloplegic refraction, optical coherence tomography (OCT), and full-field electroretinography (ERG) under the standards of the International Society for Clinical Electrophysiology of Vision. The BCVA was converted to the logarithm of the minimum angle of resolution (logMAR), where logMAR values of 0, 1.0, 2.0, and 3.0 corresponded to Snellen visual acuity measurements of 1.0, 0.1, counting fingers, and hand movements, respectively (Lange et al., 2008; Schulze-Bonsel et al., 2006). In addition, the individuals underwent initial comprehensive phenotyping evaluations, which constituted their baseline timepoints for the longitudinal analyses.

### Data analysis

Statistical Package for the Social Sciences software for Windows (version 22.0, IBM Corporation) was used for the statistical analyses. The OCT layer thicknesses and ERG amplitudes served as the primary outcome measures, while the BCVA and spherical equivalent outcomes provided secondary metrics. The continuous data were presented as mean ± standard deviation (SD) values with 95% confidence intervals (CIs), and the Friedman test was used to determine significant differences between two groups; *p* < 0.05 was considered to be statistically significant.

## Results

### Verification of a novel *RP2* mutation

**FIGURE 1:**
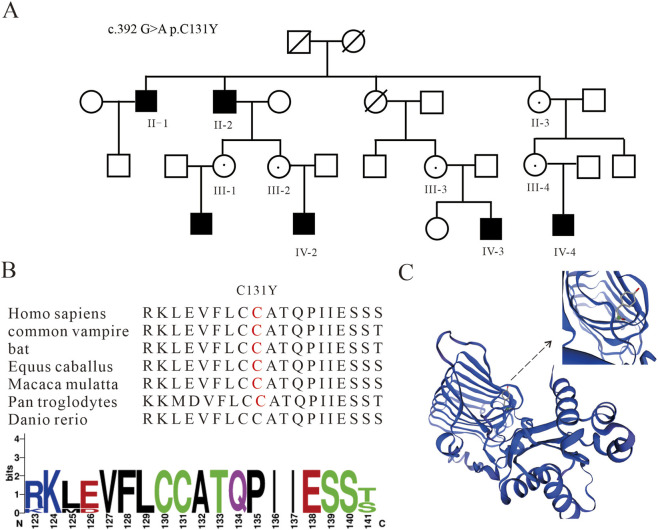
Identification of *RP2* gene mutations and locations of the variants within the structure. **(A)** Pedigree of a Chinese family comprising 23 individuals with X-linked retinitis pigmentosa (XLRP) and segregation of the *RP2* mutations identified in the members; the affected males are represented by black squares, while the genetically tested females and obligate carriers are indicated by circles with dots. **(B)** Map depicting the evolutionary conservation of amino acid residues corresponding to cysteine 131 across diverse species; multiple sequence alignment was performed using orthologous protein sequences, and the conservation score for each position is visually represented by the color intensity, with the highly conserved residues shown in darker shades. **(C)** Schematic model of the C131Y mutation-induced protein misfunction in the *RP2* gene; substitution of cysteine with tyrosine at residue 131 introduces a bulky and aromatic side chain.

We used WES to identify a novel hemizygous missense variant c.392G>A (p.C131Y) of the *RP2* gene (NP_008846.2); this variant was characterized by a cytosine to adenine substitution at the nucleotide position c.392G>A, which resulted in the replacement of a conserved cysteine residue with tyrosine at codon 131 (p.C131Y) (Supplementary Figure S1). The variant interpretation followed the ACMG guidelines. Segregation analysis across three generations demonstrated X-linked recessive inheritance, where the variant was fully cosegregated with the disease phenotype (Figure 1). Protein conservation analysis revealed that the position 131 was highly conserved across species. Meanwhile, protein structural modeling showed that the variant potentially disrupted β2 folding stability and probably impaired the interactive activities of the proteins (Figure 1; Supplementary Table S1) (Liu et al., 2017).

### Clinical presentations of the affected male subjects and female carriers

Among the 23 participating individuals, six males were identified as affected patients with hemizygous mutations and five females were eventually confirmed as carriers with heterozygous mutations. The six affected male patients exhibited consistent yet variable phenotypic manifestations at baseline evaluation. However, owing to the fact that II-1 and II-2 experienced severe retinal detachment, vitreous hemorrhage with cataract, and lacked effective medical intervention, they only retained slight vision for light perception and could not be examined comprehensively (Table 1).

**TABLE 1:** Clinical characteristics of individuals with X-linked retinitis pigmentosa (XLRP) in a Chinese family.

Pedigree Number	Age at baseline (years)	Type	BCVA	Diopter	FP	OCT	ERG	Complications
II-1	67	Mutated	LP/LP	NA	NA	NA	NA	Cataract, RD, and VRH in both eyes
II-2	64	Mutated	LP/LP	NA	NA	NA	NA	Cataract and RD in both eyes
II-3	54	Carried	0.6/0.8	OD: +0.25D/–0.50D×180 OS: +1.00D/–0.50D×180	Pathologic myopic (PM) changes in both eyes	Relatively normal	Decreasing amplitudes of a and b waves	None
III-1	36	Carried	0.2/0.6	OD: –4.25D/–0.25D×11 OS: –3.75D/–0.750D×185	Hyperpigmented deposits, attenuated vessels, and PM changes of the OD; relatively normal OS	Disordered outer retinal bands and macular atrophy in the right eye	Decreasing amplitudes of a and b waves	None
III-2	32	Carried	0.8/1.0	OD: +0.75D/–1.25DC×90 OS: +1.25D	Relatively normal	Slightly disordered outer retinal bands in the peripheral retina	Relatively normal	None
III-3	29	Carried	0.3/0.4	OD: –2.75D/–1.75D×10 OS: –3.00D/–1.75D×170	Hyperpigmented deposits, attenuated vessels, and retinal atrophy	Disordered outer retinal bands	Decreasing amplitudes of a and b waves	None
III-4	25	Carried	0.3/0.3	OD: –2.50D/–0.50D×10 OS: –1.50D/+1.0D×80	Optic disc pallor, attenuated vessels, and retinal atrophy	Posterior vitreous detachment and degeneration of the outer retinal layers	Significantly decreasing amplitudes	None
IV-1	1	Mutated	NA/NA	OD: –4.75D/–0.75DC×10 OS: –5.25D/–1.0DC×121	Pigmentary changes and tessellated fundus	Thinning stratification of the outer layers	Significantly decreasing amplitudes	None
IV-2	4	Mutated	0.4/0.3	OD: –2.75D/–1.50D×35 OS: –2.75D/–1.50D×165	Hyperpigmented deposits and macular atrophy	Thinning of the outer retinal layers	Decreasing amplitudes	None
IV-3	8	Mutated	0.2/0.2	OD: –2.25D/–2.50D×180 OS: –2.50D/–1.50D×11	Widespread hyperpigmentation and retinal atrophy	Degeneration of the outer retinal layers	Decreasing amplitudes of a and b waves	None
IV-4	6	Mutated	0.5/0.6	OD: –3.50D/–0.75D×5 OS: –4.50D/–1.50D×170	Tessellated fundus and hyperpigmentation deposits	Disordered outer retinal bands	Decreasing amplitudes	None

OD, right eye; OS, left eye; DC, cylinder diopter; BCVA, best-corrected visual acuity; NA, not available; ERG, electroretinography; FP: fundus photograph; OCT, optical coherence tomography; LP: light perception; RD, retinal detachment; VRH, vitreous hemorrhage. The age values reflect patient ages during the first comprehensive assessment (baseline) in this study, whereas disease onset occurred prior to baseline.

**FIGURE 2:**
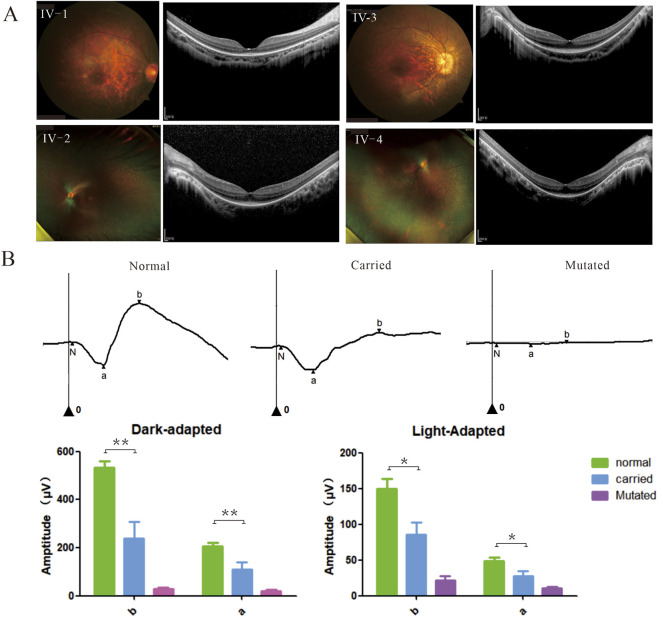
Clinical presentation of the affected male subjects. **(A)** Direct fundus of IV-1 indicates hyperpigmented deposits, tessellated changes, and retinal vessel rigidity; patient IV-2 shows hyperpigmented deposits, tessellated changes, and swelling of the optic disc in the fundus; fundus photograph of patient IV-3 shows widespread hyperpigmentation and retinal atrophy; patient IV-4 shows hyperpigmented deposits, tessellated changes, and yellowish-white punctate exudation in the retina. The optical coherence tomography (OCT) images of all patients indicate thinning of the outer retinal layers. **(B)** Electroretinography (ERG) of the affected and carrier individuals. The amplitudes of the a-wave and b-wave for five normal subjects, five carrier individuals (II-3, III-1, III-2, III-3, and III-4, n = 5), and four affected individuals (IV-1, IV-2, IV-3, and IV-4, n = 4). **p < 0.01, *p < 0.05.

The proband IV-1 was a 1-year-old child who was identified with poor visual acuity and moderate myopia (oculus dexter or right eye: −4.75D/–0.75DC×10; oculus sinister or left eye: −5.25D/–1.0DC×121). Further, a tessellated fundus and mild hyperpigmented deposits in the retina were revealed through a RetCam examination. Structural evaluation using OCT revealed degeneration and loss of the outer retinal bands in the peripheral retina, particularly in the ellipsoid zone, while the relative structure was preserved and central macula was spared. During the initial evaluations, the amplitudes of the a-wave and b-wave were found to be drastically reduced compared to those of normal individuals. Similarly, identical mutations were confirmed in the other patients (II-1, II-2, IV-2, IV-3, and IV-4), all of whom exhibited typical manifestations of RP (Table 1). BCVA measurements revealed significant impairments with a mean logMAR value of 0.5235 (range: 0.301–0.698), indicating moderate visual dysfunction across the cohort. Refractive assessments demonstrated a trend toward moderate myopia with an average spherical equivalent (SE) of −4.15D (range: −3.125D to −5.125D). The fundus images revealed pigmentary abnormalities (100%, 8/8 eyes), tessellated changes (75%, 6/8 eyes), swelling of the optic disc (12.5%, 1/8 eyes), and yellowish-white punctate exudation (50%, 4/8 eyes). OCT demonstrated uniform disorganization of the outer retinal layers, particularly disruption of the outer layer, consistent with the typical rod-cone dystrophy pattern observed in RP (Figure 2; Supplementary Figure S2). Moreover, functional assessments through ERG showed detectable and statistically significant reductions in both the a-wave (average: 20.90 ± 17.05 μV and 10.76 ± 6.50 μV) and b-wave (average: 28.33 ± 21.59 μV and 22.01 ± 16.58 μV) amplitudes under dark and light adaptation conditions compared to age-matched controls (a-wave average: 206.45 ± 44.98 μV and 48.84 ± 13.76 μV, b-wave average: 531.85 ± 90.44 μV and 150.00 ± 43.98 μV) (all *p* < 0.001, Figure 2). The preserved yet subnormal ERG responses suggest partial retention of photoreceptor and bipolar cell functions during the early stages of the disease despite the structural abnormalities (Wu et al., 2023).

**FIGURE 3:**
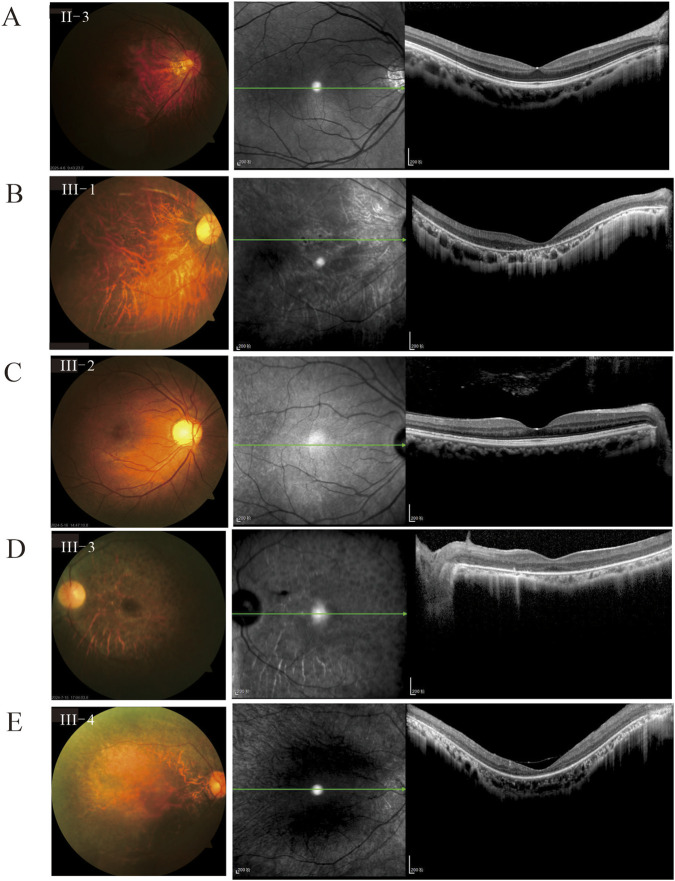
Color fundus photographs and OCT images of the female carriers. **(A)** Fundus of II-3 shows tessellated changes in the retina and relatively normal layers in the OCT image. **(B)** Fundus of III-1 shows retinal vessel rigidity, tessellated changes, and retinal atrophy, where the OCT image shows atrophy of the entire retina. **(C)** Fundus of III-2 and normal OCT image. **(D)** Fundus of III-3 shows widespread hyperpigmented deposits and optic disc pallor; the OCT image shows atrophy of the entire retina and foveal schisis changes. **(E)** Fundus of III-4 shows retinal atrophy with attenuated and straightened retinal vessels; the OCT image shows atrophy of all retinal layers and posterior vitreous detachment.

The heterozygous female carriers [II-3, III-1, III-2, III-3, and III-4; average age: 35.5 (25–54) years] presented a relatively severe phenotypic spectrum at baseline evaluation (Figure 3; Table 1). Only one carrier (III-2) showed relatively normal visual function and retinal structure, while the others demonstrated significant pathologies (Figure 3; Supplementary Figure S3). Among the carriers, 80% (8/10 eyes) showed poor BCVA (logMAR 0.397–0.097) and 70% (7/10 eyes) suffered from myopia (average: −1.75DS, range: −4.375DS to 1.25DS). Notably, the proportion of moderate myopia was as high as 50% (5/10 eyes). Similar to the male patients, the carriers mainly showed tessellated fundus changes (7/10 eyes), hyperpigmented deposits (6/10 eyes), attenuated vessels (6/10 eyes), and retinal atrophy (2/10 eyes). Notably, fundus examination of III-1 revealed bone spicule pigmentation and leopard changes in the posterior pole of the right eye, along with an almost normal retinal appearance in the left eye (Supplementary Figure S3). Focal outer retinal layer disruptions were identified in 7/10 eyes (70%). ERG abnormalities were also noted in 8/10 eyes (80%), with the average amplitudes of the a and b waves being 109.84 ± 92.09 μV and 239.38 ± 217.19 μV under dark-adapted conditions as well as 27.45 ± 22.39 μV and 85.17 ± 56.35 μV under light-adapted conditions, respectively (Figures 2, 3). Three carriers exhibited phenotypes approaching the severity of the affected males, which challenges the conventional diagnostic paradigms of X-linked disorders; this variability exceeds the traditional expectations for XLRP carriers and reinforces the broad spectrum of disease expression in heterozygous females (Souied et al., 1997).

### Longitudinal progression

**FIGURE 4:**
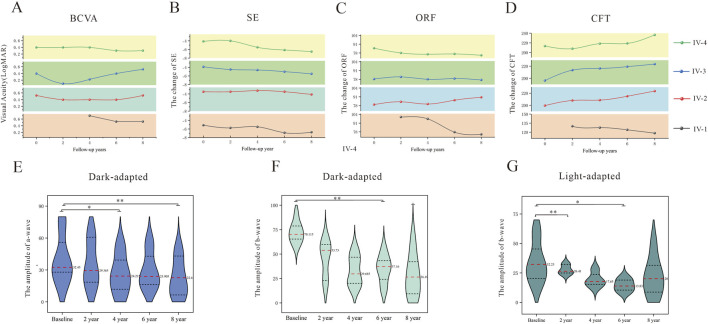
Eight-year follow-up data of the affected subjects showing biennial changes in the **(A)** best-corrected visual acuity (BCVA) in terms of the logMAR values, **(B)** spherical equivalent (SE), **(C)** outer retinal layer features (ORF), and **(D)** central foveal thickness (CFT). Electroretinography results of the affected subjects at 0, 2, 4, 6, and 8 years follow-up showing average changes to the **(E)** a-wave and **(F)** b-wave amplitudes in response to scotopic 3.0 condition and **(G)** b-wave amplitudes in response to photopic 3.0 condition. *p < 0.05, **p < 0.01.

Tracking disease progression in the subjects over a period of 8 years revealed measurable changes across multiple phenotypic parameters in the affected males; unfortunately, the other carriers except III-1 were unable to undergo regular follow-ups owing to various reasons (Supplementary Figures S4, S5). All longitudinal analyses considered the age at baseline evaluation as the temporal reference point, which was designated as the initial phenotyping visit. In general, the initial visual acuity remained almost stable or showed an increasing trend but still remained below the lowest limit of the age-standardized BCVA (Supplementary Table S2); this progression exceeds the typical age-related vision improvement, potentially confirming the degenerative nature of *RP2*-associated XLRP. The refractive measurements demonstrated concurrent worsening, with the SE shifting toward more severe myopia at the average rate of 0.28D, 0.31D, 0.65D, and 0.34D for each 2-year-period separately (Figure 4; Supplementary Table S3). Lastly, pigmentary abnormalities, tessellated changes, and yellowish-white punctate exudation were still observed in the fundus, which did not differ significantly from the baseline.

During the follow-up period, structural assessments via OCT imaging were used to quantify progressive degeneration of the retinal layer. Over the course of the 8-year follow-up, the outer retinal layers of IV-2 increased (2.7 μm/2 years) at the fourth year, while those of the other three patients decreased at the rate of 8.55 μm, 0.33 μm, and 2.625 μm per 2 years; among these, the decrease noted for IV-1 was the sharpest. Paradoxically, the central foveal thickness exhibited slight increases of 6.04 μm, 6.05μm, and 5.75 μm per 2 years for three of the patients, while IV-1 showed a decline of 3.83 μm per 2 years. The dissociation changes highlight the regional variability in disease progression, with the central regions demonstrating rapid degeneration (Supplementary Tables S4, S5). Over the follow-up period, the average amplitudes of the a-wave under dark-adapted conditions at 0, 2, 4, 6, and 8 years were 40.44 ± 23.39 μV, 35.67 ± 21.32 μV, 25.91 ± 18.18 μV, 29.33 ± 17.10 μV, and 22.60 ± 17.89 μV, while the amplitudes of the b-wave were 72.26 ± 10.90 μV, 45.24 ± 18.10 μV, 33.12 ± 14.92 μV, 34.85 ± 14.10 μV, and 28.31 ± 21.55 μV, respectively. In addition, the b-wave amplitudes under light-adapted conditions at 0, 2, 4, 6, and 8 years were 34.23 ± 17.16 μV, 27.77 ± 3.96 μV, 19.26 ± 6.09 μV, 14.59 ± 5.22 μV, and 22.01 ± 16.58 μV, respectively (Supplementary Table S6). The ERG documented progressive functional decline, with the differences of the cone- and rod-mediated responses deteriorating in a statistically significant manner (Verbakel et al., 2018). This differential vulnerability is aligned with the known natural history of XLRP, where cone dysfunction typically follows rod degeneration (Wensel et al., 2021; Zada et al., 2021). The b-wave amplitudes showed greater reductions than the a-wave amplitudes, which suggests progressive inner retinal involvement over time. Moreover, during the 8-year follow-up period, none of the four young patients experienced significant complications.

## Discussion

In this study, we identified a novel variant c.392G>A of the *RP2* gene in a Chinese pedigree including six patients and five female carriers. To the best of our knowledge, this is the first report based on detailed ophthalmological examinations of these individuals along with an 8-year clinical follow-up period for the male patients. This initiative expands the pathogenic spectrum of disease mutations as well as enriches the clinical phenotypes and natural history of the disease. Human *RP2* encodes a ubiquitously expressed polypeptide of 350 amino acids containing an N-terminal tubulin folding cofactor C-like (TBCC) domain and a C-terminal nucleoside diphosphate kinase-like domain (Liu et al., 2017). To date, more than 200 pathogenic mutations have been identified in *RP2*. According to previous literature, approximately two-thirds of these mutations are predicted to result in a truncated C-terminal portion of the RP2 protein or complete loss of the *RP2* gene.

In our study, the missense variant C131Y was identified at the location of the TBCC domain, which is essential for ciliary trafficking and could potentially directly impact the affinity of RP2 and ADP-ribosylation factor like GTPase 3. Meanwhile, the variant occurs at a mutational hotspot region, where frameshift and non-sense mutations are more prevalent than missense mutations in XLRP (Koyanagi et al., 2019). Usually, frameshift and truncating mutations cause complete loss of function, while the missense variants often cause partial dysfunction or dominant negative effects (Gao et al., 2019). Notably, in this investigation, the phenotypic manifestations resulting from this variant exhibited serious structural and functional impairments, leading to clinical phenotypic variability. Although the specific pathogenic mechanisms remain unclear, we hypothesize that this phenotypic difference may be related to X-linked inheritance patterns, genetic modifiers, regulation of other genes and environmental factors, and individual variations (Thompson et al., 2020). Additionally, our discovery offers significant insights into both the clinical diagnosis of XLRP and molecular pathways behind retinal degeneration, while broadening the scope of XLRP mutations and exposing a potential molecular target for prospective treatment approaches via gene therapy (Lam et al., 2024).

Theoretically, earlier research reports show that affected males with prematurely halted translations exhibit more severe clinical symptoms than individuals with missense mutations (Lam et al., 2024; Silva et al., 2021). The proband in this study, namely a 1-year-old boy with atypical RP clinical phenotype, was diagnosed with moderate myopia and pathologic myopia of the fundus. Additionally, the other young patients (with the exception of the two older patients) exhibited serious complications, poor vision, as well as characteristic fundus observations of hyperpigmented deposits, attenuated vessels, and retinal atrophy. We simultaneously observed that two of the patients had substantial yellowish-white punctate exudations of the fundus, which are characteristic changes beyond typical retinal pigmentary changes. These presentations demonstrate that RP causes more significant visual impairments with earlier onset and more severe symptoms, particularly in infants and young children.

Research has shown that severe myopia is present in almost half of *RP2*-gene-related RP. Early-onset myopia is one of the first signs of retinal dystrophy, for which researchers usually tend to focus on protopathy while sometimes ignoring the changes in myopia (Yassin et al., 2025; Iorio et al., 2020). Thus, we considered an 8-year period of refractive evaluations, whose results demonstrate that the degree of myopia worsens gradually and that there is slight deterioration over time, suggesting that retinal degeneration may be closely related to the occurrence of myopia but has a relatively smaller impact on the development of myopia. Structural evaluations via OCT showed distortion of the outer retinal layers while preserving the relative structure and macular sparing; the distortions mainly consisted of the typical rod–cone dystrophy pattern (Gao et al., 2019; Iorio et al., 2020). According to a study, the outer nuclear layer thinned at a rate of 2.52–2.58 μm annually during the 3-year follow-up observation period; however, the progression rate observed via OCT slowed by 1.7 μm annually, which may be attributed to the relatively youthful patients and duration of the follow-up period (Zada et al., 2021). Further, the contradictory alterations in the ocular retinal layer and central foveal thickness, which are probably caused by Muller cell gliosis, underscore the difficulty of deciphering the structural alterations in degenerative retinopathies and emphasize the significance of multimodal imaging in clinical evaluations (Claire et al., 2015).

In terms of the ERG data, the amplitudes of the a and b waves showed significant reductions during the early stages of disease diagnosis (Verbakel et al., 2018); Although there was no statistically significant difference, the response rate exhibited a declining trend over the 8-year follow-up period. Despite clear structural abnormalities, the preserved responses indicate partial preservation of the photoreceptor and bipolar cell functions, even sustaining the relative stability in the early stages of the disease. This dissociation between the structural and functional measures could reflect the compensatory mechanisms or variable disease expression among affected individuals. The quantified rates of BCVA loss, outer retinal thinning, and ERG amplitude reductions provide concrete benchmarks for therapeutic development (Kong et al., 2025; Votruba et al., 1998). In most cases, the accelerated functional decline observed after the age of 30 years suggests that therapeutic interventions may be most effective (Cehajic-Kapetanovic et al., 2020). However, the progressive declines in retinal structure and visual function observed in this study could highlight the necessity for therapeutic interventions earlier in the course of the disease.

According to previous reports, heterozygous female carriers of XLRP showed a wide range of functions and usually had mildly or moderately reduced visual functions. Further, most cases exhibited distinct degrees of fundus changes with bilateral symmetry. Nevertheless, compared to other carriers with other XLRP variant sites, carriers with this specific site have been noted to be more prone to ocular abnormalities and even exhibit more severe phenotypes (Souied et al., 1997). One study argues that XLRP patients exhibiting different phenotypes can be explained as a chimera resulting from lyonization or random X-inactivation (Silva et al., 2021; Gocuk et al., 2024); this phenomenon is popularly known to be caused by chromosomal disjunction, asymmetric mitosis, gametic half-chromatid variations, mitotic selective chromatid segregation, and post-zygotic mutations. More intriguingly, our study presents the multimodal imaging characteristics of a female carrier exhibiting remarkable asymmetry between the eyes. We acknowledge that no direct X-chromosome inactivation skewing assays were performed in our cohort. Thus, we hypothesize that the proportion of mutant X-chromosomes would be greater in the right eye than the left eye at the mosaic embryonic stage. However, modifier genes and environmental influences are also potential factors, for which more research is needed. The broad phenotypic spectrum among female carriers challenges traditional diagnosis and has immediate implications for genetic counseling (Comander et al., 2015). Regrettably, the limited follow-up data made us realize that all female carriers should have thorough retinal evaluations and long-term observations. Additionally, the identification of progressive carriers could argue for their inclusion in natural history studies and clinical trials, so that they may benefit from therapeutic interventions (Clark et al., 2025; Gocuk et al., 2024).

Notwithstanding the findings reported herein, we acknowledge that there are certain limitations to this study, including a small cohort size, absence of longitudinal data on female carriers, and knowledge of the underlying biological mechanisms. The relatively small sample size, particularly in a longitudinal cohort, could limit the generalizability of the progression rate. Further, our focus on a single pedigree in this study restricts the ability to assess population-level variability in disease expression and even lacks functional validation. These limitations highlight the need for larger and multicenter studies to validate the observed progression patterns and to explore potential modifying factors, such as environmental influences or genetic background effects (Ford and Petersen-Jones, 2025).

In summary, we report the detection of a novel mutation c.392G>A of the *RP2* gene in a Chinese pedigree and elucidate the natural history of four affected males with XLRP during the 8-year follow-up period; we thus demonstrate that the p.C131Y variant causes progressive retinal degeneration with measurable declines in visual function and structural integrity. The XLRP carriers in this study showed a wide range of phenotypic variability, and most carriers had mildly or moderately reduced visual functions. Intriguingly, one female carrier showed marked asymmetric retinal involvement. Our longitudinal data establish some critical benchmarks for disease progression to inform clinical trial design and therapeutic development in the future. Future research efforts should explore the molecular mechanisms underlying the observed phenotypic heterogeneity and investigate targeted interventions for this specific variant, along with management strategies for the XLRP patient.

## Data Availability

The datasets presented in this study can be found in online repositories. The names of the repositories and accession numbers can be found in the article/Supplementary Material.
