# Supplementary material for: Eight-year follow-up of phenotypic progression in a Chinese XLRP pedigree with a novel RP2 gene mutation
Source: Front Genet. 2026 May 25;17:1783270. doi: 10.3389/fgene.2026.1783270 (PMC13242896; doi:10.3389/fgene.2026.1783270)
Supplement: Supplementary file 1 [file DataSheet1.doc]

**Eight-year Follow-up of Phenotypic Progression in a Chinese XLRP Pedigree with a Novel *RP2* Gene Mutation**

**Supplemental information files**

**Supplemental Figures**

**Supplemental Tables**

**Supplemental Methods**

**Supplemental Figures**


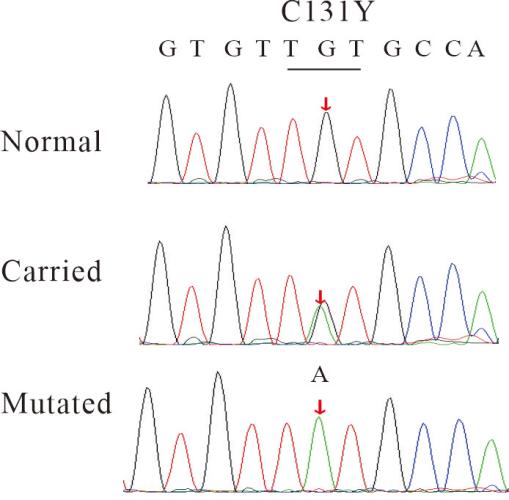


**Supplemental Figure 1**. Sanger sequence of *RP2* of participants in a Chinese family Vertical arrows indicate the mutation site in sequencing chromatograms. The mutaion (c. 392 G>A p. C131Y ) was confirmed by sanger sequence in all patients (IV-1, IV-1, IV-3, and IV-4) and heterozygou variant in carriers (II-3, III-1, III-2, III-3, III-4).


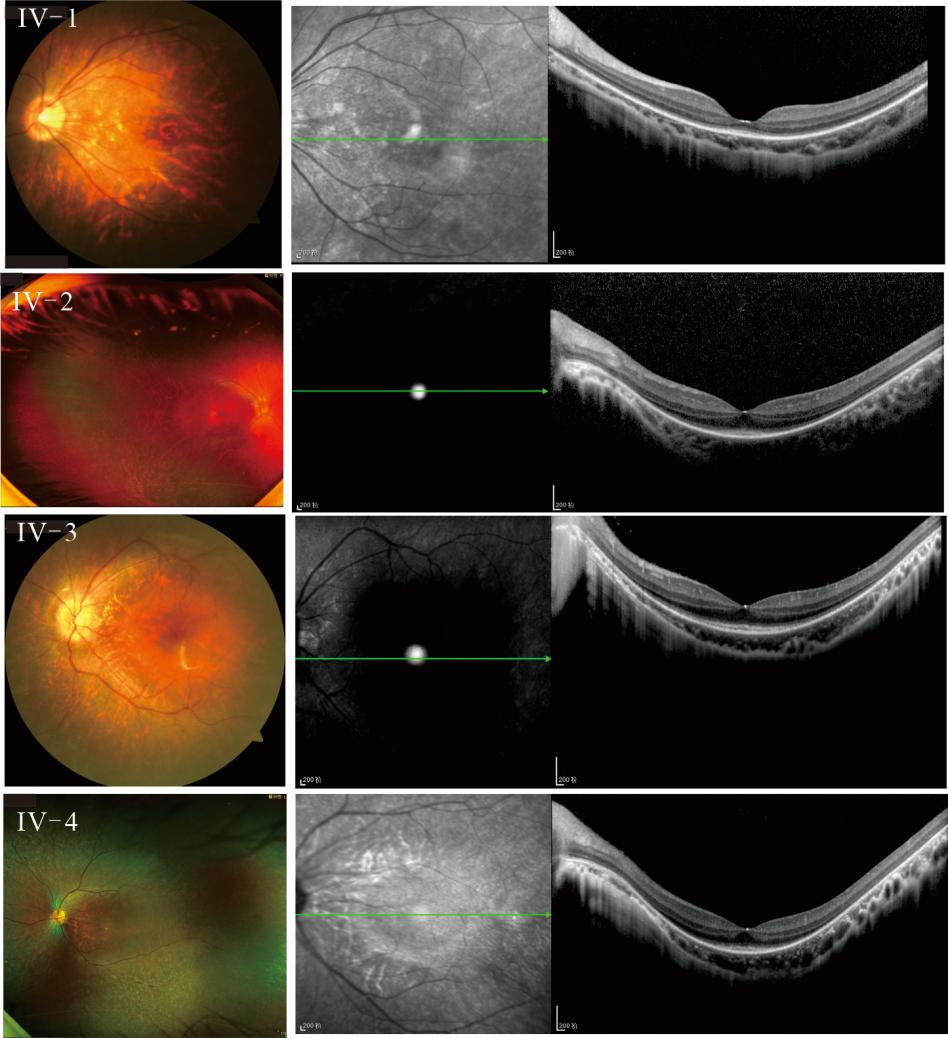


**Supplemental Figure 2. Fundus photographs and optical coherence tomography (OCT) image in affected subjects.** (A) The fundu of IV-1’s left eye indicate tessellated changes, as well as retinal vessel rigidity; OCT reveal the thinning of outer layers in retina. (B) The fundu of IV-2’s right eye indicate tessellated changes and the thinning of outer layers in retina of OCT; (C) The fundu of IV-3’s left eye indicate tessellated changes and the swelling of the optic disc; OCT reveal the thinning of outer layers in retina. (D) The fundu of IV-3’s left eye indicate tessellated changes as well as yellowish-white punctate exudation in peripheral retina. OCT reveal the thinning of outer layers in retina.


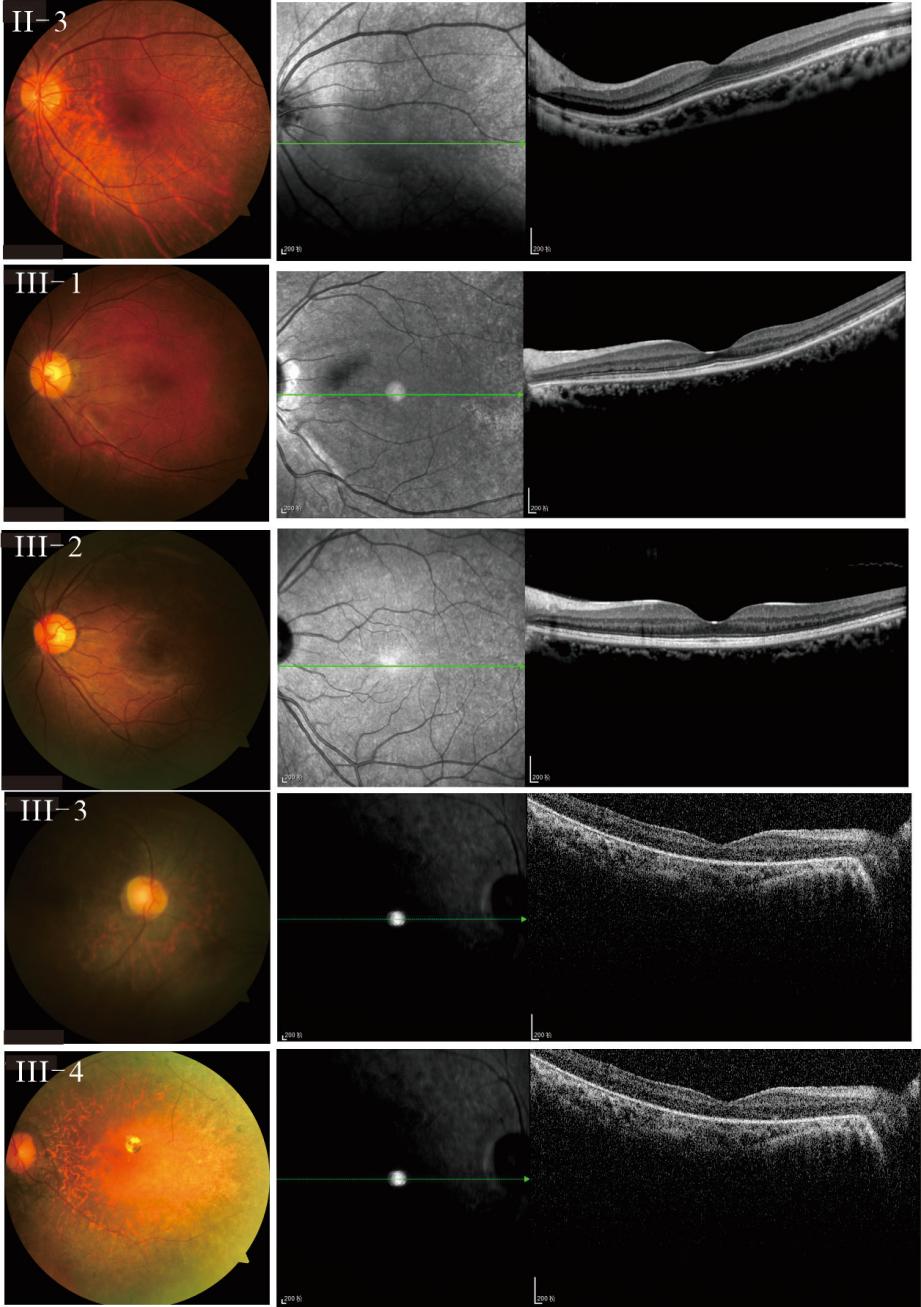


**Supplemental Figure 3. Fundus photographs and optical coherence tomography (OCT) image of carriers.** (A) The fundu of II-3’s left eye indicate tessellated changes; OCT reveal the thinning of outer layers in retina. (B) The fundu of III-1’s left eye display normal presentation. OCT detected the disorder and thinning in peripheral retina; (C) III-2’s fundus and OCT performed normal; (D) The fundu of II-3’s right eye showed widespread retinal atrophy and optic disc pallor. OCT detected the atrophy in whole retina; (E) The fundu of II-3’s left reveal retinal atrophy, straightened retinal vessels. OCT showed whole atrophy of retina.


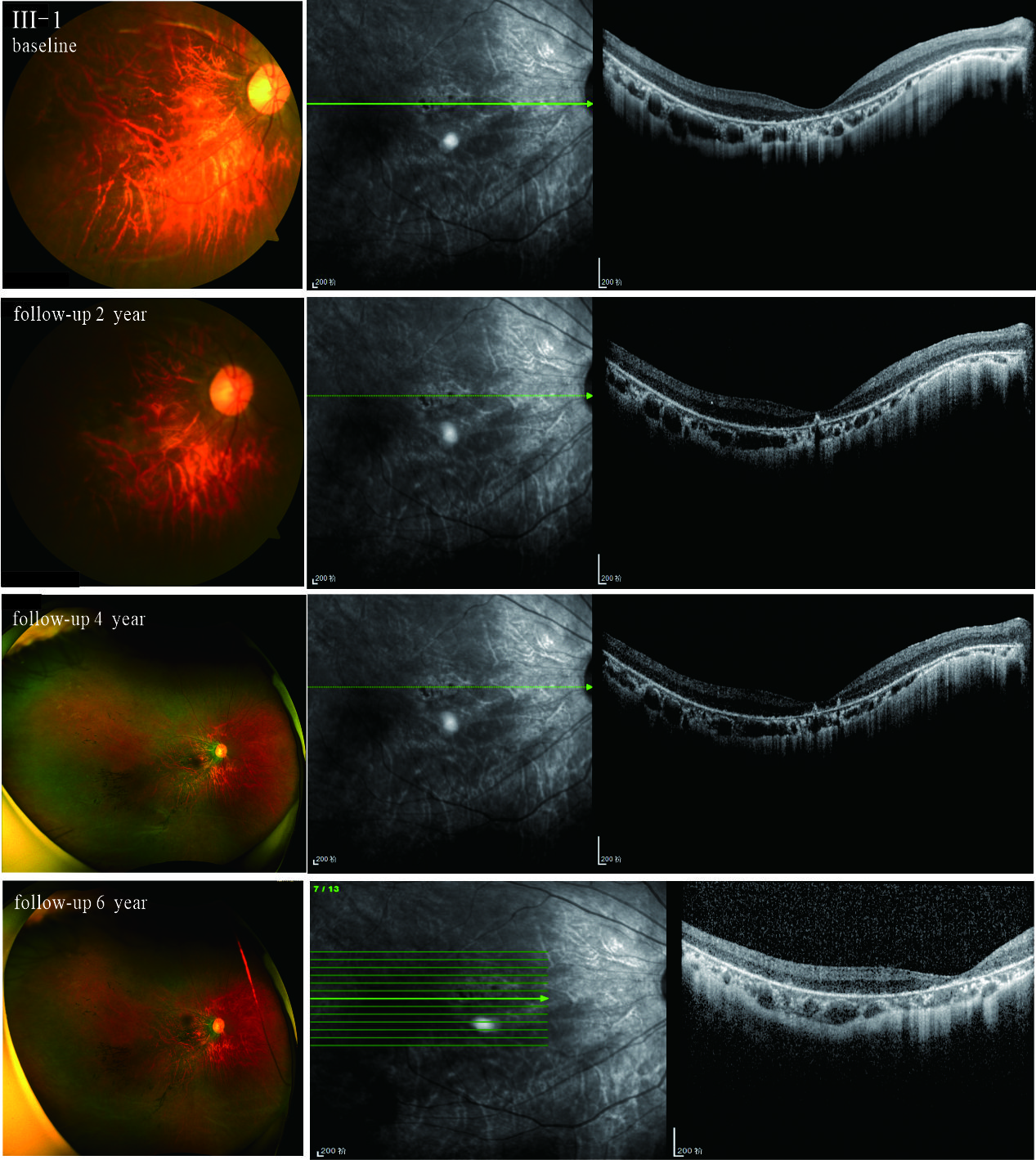


**Supplemental Figure 4. Fundus photographs and optical coherence tomography (OCT) image of III-1 with 6 years follow up.**

**
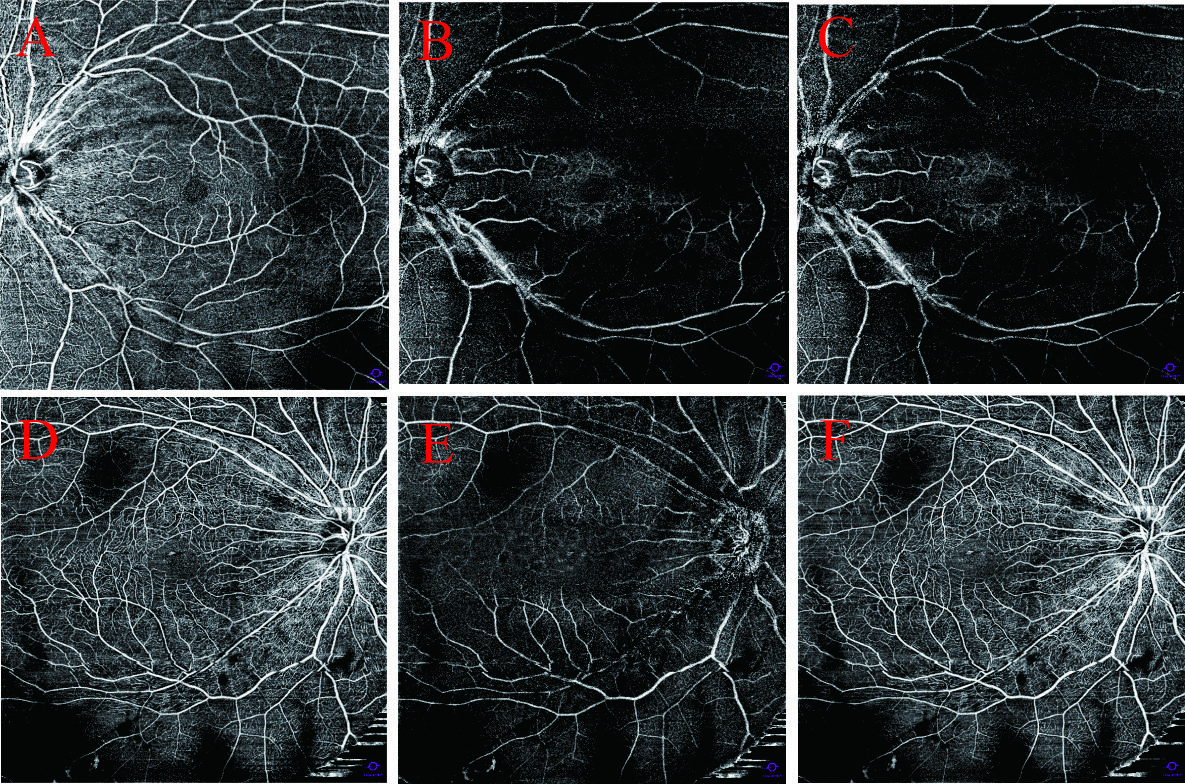
**

**Supplemental Figure 5. Optical coherence tomography angiography images (12×12 mm)**. These correspond to superficial capillary plexus (A, D) deep capillary plexus slab (B, E) and retinal thickness map (C, F).

**Supplemental table 1. Structural and phylogenetic Evidence for pathogenicity of the variant.**

| **Prediction Tool** | **Category** | **Prediction** | **Support Strength** |
| --- | --- | --- | --- |
| REVEL | Ensemble pathogenicity | Damaging (0.98) | Strong |
| PolyPhen-2 | Functional impact | Damaging (0.994) | Highest confidence |
| SIFT | Functional impact | Damaging (0.001) | Strong |
| MutationTaster | Disease probability | Disease-causing (1) | high probability |
| MCAP | Disease probability | Pathogenic(0.2542) | Strong |
| SPIDEX | Disease probability | -0.081 | - |
| dbscSNV | - | - | - |
| spliceAI | - | - | - |
| Validation primers(F) | TTTCTGGGACCCGTGAAAGG | | |
| Validation primers(R) | AGTCCTGAACCACAGCATCT | | |

| **N0.** | **Type** | **0 year** | **2 years** | **4 years** | **6 years** | **8 years** |
| --- | --- | --- | --- | --- | --- | --- |
| IV-1 | R | NA | NA | 0.699 | 0.523 | 0.523 |
|  | L | NA | NA | 0.699 | 0.699 | 0.523 |
| IV-2 | R | 0.523 | 0.397 | 0.397 | 0.397 | 0.523 |
|  | L | 0.523 | 0.397 | 0.397 | 0.397 | 0.397 |
| IV-3 | R | 0.397 | 0.097 | 0.222 | 0.397 | 0.523 |
|  | L | 0.523 | 0.301 | 0.301 | 0.523 | 0.523 |
| IV-4 | R | 0.397 | 0.397 | 0.397 | 0.301 | 0.301 |
|  | L | 0.397 | 0.397 | 0.301 | 0.301 | 0.222 |

**Supplemental table 2. Summary of best-corrected visual acuity (logMAR) with follow up.**

Note: R right eye; L left eye;

**Table 3. Summary of spherical equivalent (D) with follow up.**

| **N0.** | **Type** | **0 year** | **2 years** | **4 years** | **6 years** | **8 years** |
| --- | --- | --- | --- | --- | --- | --- |
| IV-1 | R | -5.12 | -5.75 | -5.50 | -6.875 | -6.75 |
|  | L | -5.75 | -6.875 | -6.75 | -7.50 | -7.875 |
| IV-2 | R | -3.50 | -3.50 | -3.25 | -3.50 | -4.125 |
|  | L | -3.50 | -3.625 | -3.625 | -3.375 | -3.875 |
| IV-3 | R | -3.875 | -4.50 | -4.625 | -5.00 | -5.50 |
|  | L | -4.125 | -4.375 | -4.625 | -4.75 | -5.75 |
| IV-4 | R | -4.125 | -4.00 | -5.50 | -6.125 | -6.50 |
|  | L | -5.25 | -5.25 | -6.25 | -6.875 | -7.00 |

Note: R right eye; L left eye;

**Table 4. Summary of the average central foveal thickness with follow up.**

| **N0.** | **Type** | **0 year** | **2 years** | **4 years** | **6 years** | **8 years** |
| --- | --- | --- | --- | --- | --- | --- |
| IV-1 | R | NA | 131 | 124 | 123 | 119 |
|  | L | NA | 120 | 132 | 121 | 118 |
| IV-2 | R | 198 | 205 | 213 | 216 | 222 |
|  | L | 197 | 205 | 212 | 222 | 221 |
| IV-3 | R | 206 | 214 | 218 | 217 | 223 |
|  | L | 210 | 216 | 224 | 227 | 250 |
| IV-4 | R | 213 | 208 | 217 | 223 | 236 |
|  | L | 215 | 208 | 212 | 222 | 228 |

Note: R right eye; L left eye;

**Table 5. Summary of outer average retinal layer with follow up.**

| **N0.** | **Type** | **0 year** | **2 years** | **4 years** | **6 years** | **8 years** |
| --- | --- | --- | --- | --- | --- | --- |
| IV-1 | R | NA | 100 | 97 | 75 | 75 |
|  | L | NA | 103 | 96 | 75 | 72 |
| IV-2 | R | 78 | 81 | 84 | 85 | 83 |
|  | L | 76 | 81 | 80 | 81 | 80 |
| IV-3 | R | 80 | 82 | 74 | 78 | 77 |
|  | L | 84 | 81 | 84 | 79 | 76 |
| IV-4 | R | 85 | 78 | 76 | 75 | 75 |
|  | L | 83 | 80 | 80 | 75 | 74 |

**Table 6. Summary of amplitude of ERG with follow up.**

| amptitude | **0 year** | **2 years** | **4 years** | **6 years** | **8 years** |
| --- | --- | --- | --- | --- | --- |
| a-wave  In dark-adatped | 40.44 ± 23.39μV | 35.67 ± 21.32μV | 25.91 ± 18.18μV | 29.33 ± 17.10μV | 22.60 ± 17.89μV |
| b-wave  In dark-adatped | 72.26 ± 10.90μV | 45.24 ± 18.10μV | 33.12 ± 14.92μV | 34.85 ± 14.10μV | 28.31 ± 21.55μV |
| b-wave  In light-adatped | 34.23 ± 17.16μV | 27.77 ± 3.96μV | 19.26 ± 6.09μV | 14.59 ±5.22μV | 22.01 ± 16.58μV |

1. Fundus Photography (Retinography)

Device: Hotline retinal camera

Manufacturer: Gaoshi Medical Equipment Co.

Country: China

Parameters:

Field of view: 45°

Image resolution: 2048 × 2048 pixels

Illumination: white LED

Pupillary dilation: tropicamide (minimum pupil diameter 6 mm)

Exposure: auto mode, manually adjusted if needed

Quality check: no motion artifact, proper foveal centration

2. Full-Field Electroretinography (ERG)

Device: ROLAND CONSULT RETI-PORT/SCAN 21

Manufacturer: Roland Consult GmbH

Country: Germany

Procedures:

Performed in accordance with ISCEV standards.

Dark adaptation: 20 min

Light adaptation: 10 min

Electrodes: DTL fiber active electrode; reference at outer canthus; ground on the forehead

Sampling rate: 1,000 Hz

Bandwidth: 0.3–300 Hz

Averaging: ≥3 traces per stimulus

Quality control: baseline noise <10 µV

Stimulus conditions:

Scotopic rod response (0.01 cd·s/m²)

Scotopic maximal response (3.0 cd·s/m²)

Photopic single-flash response (3.0 cd·s/m², background 30 cd/m²)

30-Hz flicker ERG (3.0 cd·s/m²)

3. Optical Coherence Tomography (OCT)

Device: Spectral-Domain OCT (Spec-TR-04852)

Manufacturer: Heidelberg Engineering GmbH

Country: Germany

Acquisition parameters:

Scan protocol: Horizontal macular cube

Number of B-scans: 25–49

Axial resolution: ~7 µm

Transverse resolution: ~14 µm

Scan length: 6 mm

ART averaging: 5–15 frames

Segmentation: Automated with manual correction

Minimum acceptable signal: ≥20 dB

Time-domain OCT (historic data): resolution 10–12 µm, scan length 6 mm

Quality Control Procedures:

All imaging and ERG were performed by two senior ophthalmologists.

Images were independently reviewed by two retina specialists.

OCT B-scan segmentation was corrected manually when necessary.

ERG recordings were repeated if noise exceeded the threshold.
